# The monosialoganglioside GM1a protects against complement attack

**DOI:** 10.1038/s41420-023-01686-6

**Published:** 2023-10-25

**Authors:** Henri Wedekind, Julia Beimdiek, Charlotte Rossdam, Elina Kats, Vanessa Wittek, Lisa Schumann, Inga Sörensen-Zender, Arno Fenske, Birgit Weinhold, Roland Schmitt, Andreas Tiede, Falk F. R. Büttner, Anja Münster-Kühnel, Markus Abeln

**Affiliations:** 1 Institute of Clinical Biochemistry, Hannover Medical School, Hannover, Germany; 2 Department of Nephrology and Hypertension, Hannover Medical School, Hannover, Germany; 3 Department of Hematology, Hemostasis, Oncology and Stem Cell Transplantation, Hannover Medical School, Hannover, Germany

**Keywords:** Glycobiology, Complement cascade, Biologics

## Abstract

The complement system is a part of the innate immune system in the fluid phase and efficiently eliminates pathogens. However, its activation requires tight regulation on the host cell surface in order not to compromise cellular viability. Previously, we showed that loss of placental cell surface sialylation in mice in vivo leads to a maternal complement attack at the fetal-maternal interface, ultimately resulting in loss of pregnancy. To gain insight into the regulatory function of sialylation in complement activation, we here generated trophoblast stem cells (TSC) devoid of sialylation, which also revealed complement sensitivity and cell death in vitro. Glycolipid-analysis by multiplexed capillary gel electrophoresis coupled to laser-induced fluorescence detection (xCGE-LIF) allowed us to identify the monosialoganglioside GM1a as a key element of cell surface complement regulation. Exogenously administered GM1a integrated into the plasma membrane of trophoblasts, substantially increased binding of complement factor H (FH) and was sufficient to protect the cells from complement attack and cell death. GM1a treatment also rescued human endothelial cells and erythrocytes from complement attack in a concentration dependent manner. Furthermore, GM1a significantly reduced complement mediated hemolysis of erythrocytes from a patient with Paroxysmal nocturnal hemoglobinuria (PNH). This study demonstrates the complement regulatory potential of exogenously administered gangliosides and paves the way for sialoglycotherapeutics as a novel substance class for membrane-targeted complement regulators.

## Introduction

The complement system is part of the innate immune system in the fluid phase and one of the first defense lines against pathogens. One mechanism by which complement activation confers protection against pathogens is the formation of pores in the cell surface, called C5b-9 (also membrane attack complex, MAC). While complement-mediated lysis represents an effective way to eliminate pathogenic entities, host cells require strict complement regulation to remain unscathed. All cells in the circulation are permanently exposed to low levels of complement activation. This includes circulating cells as well as endothelial cells and placental cells facing the maternal fluid phase. Excessive complement activation compromises host cell viability and is involved in numerous pathologic conditions [1].

Immune homeostasis at the fetal-maternal interface is particularly intricate. The placental surface facing the maternal blood represents an interface between two genetically distinct entities and needs to establish immune tolerance to the embryo, while simultaneously ensuring potent immune defense against pathogens. This task is especially challenging in placentas of humans and rodents, in which the fetal trophoblast cells are in direct contact with the fluid phase of the maternal circulation. Loss of fetal-maternal immune balance results in pregnancy complications such as preeclampsia, or in severe cases, premature termination of pregnancy. Dysregulated complement activation was identified as a common pathway of injury in different human pregnancy complications [2–4]. Recently we made use of a sialylation deficient mouse model and showed that sialylation plays a crucial role in placental complement regulation [5]. Complete loss of fetal sialoglycans resulted in activation of the alternative pathway of the maternal complement system and ultimately embryonic death.

Loss of sialic acid on the cell surface can naturally occur due to its chemical instability, e.g., during aging or storage of cells for transfusion, or by the action of neuraminidases (Neu) of endogenous or pathogenic origin [6–8]. The influence of sialoglycans on complement regulation per se has been known for a long time and can mainly be attributed to the interaction of the complement regulatory protein FH with cell surface sialoglycans. FH regulates complement activation by dismantling of C3 convertases and the recruitment of factor I, which proteolytically inactivates C3b to iC3b. Analysis of the interaction between sialoglycans and FH culminated in the crystal structure of the FH domains 19-20 with 3’sialyllactose (Neu5Acα2-6Galβ1-4Glc) [9]. Although the minimal binding motif for the sialoglycan-FH interaction has been identified, it remained elusive whether sialoglycoconjugates could be exploited as complement regulatory reagents.

Here, we generated sialylation negative TSC, which were shown to be highly complement sensitive in vitro. Incorporation of the exogenously administered ganglioside GM1a into the membrane enhanced recruitment of the complement regulatory protein FH to murine TSC. Moreover, GM1a restored complement regulation also of human endothelial cells, sheep erythrocytes and human erythrocytes from a patient with PNH.

## Results

### Generation of asialo trophoblast stem cells

**Fig. 1:**
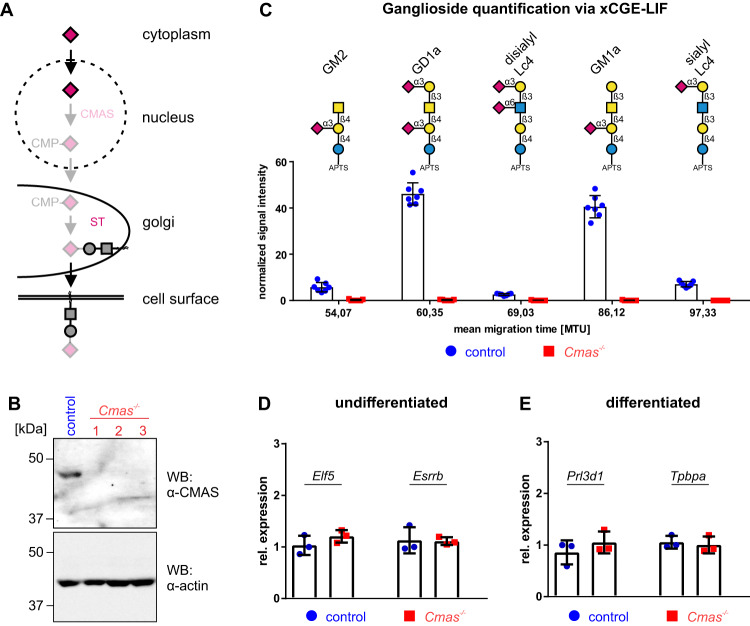
*Cmas*^*−/−*^ trophoblast stem cells are devoid of sialoglycans. **A** Scheme of the biosynthesis of sialoglycoconjugates. Genetic inactivation of *Cmas* results in unsialylated glycans at the cell surface. Sia is depicted as red diamond. **B** CMAS Western blot analysis. Control and *Cmas*^*−/−*^ TSC were lysed and separated by SDS-PAGE and immunostained with anti-CMAS antibody. Anti-actin immunostaining was used as loading control. **C** Quantification of sialylated glycans derived from glycosphingolipids of control and one representative *Cmas*^*−/−*^ TSC clone using xCGE-LIF. Glycan notations for GM2, GD1a and GM1a refer to the respective glycosphingolipid derived glycan. For inter-sample comparisons signal intensities were normalized to Man6 (nRFU), *n* = 7. MTU migration time unit. Symbols nomenclature according to [31]. Quantitative PCR of TSC markers *Elf5* and *Esrrb* (**D**) and differentiation markers *Prl3d1* and *Tpbpa* (**E**) from control and *Cmas*^*−/−*^ TSC in undifferentiated and 7 day differentiated cells in medium with heat inactivated FCS (HI-FCS). Gene expression was plotted relative to control TSC. *n* = 3. Mean values are depicted with standard deviation.

Using a sialylation deficient mouse model we previously showed, that sialoglycans are indispensable for cells of the developing placenta to ensure sufficient complement regulation [5]. In order to acquire further insights into the mechanism of placental complement regulation we made use of TSC and established a sialylation deficient TSC line by targeting the Sia-activating enzyme CMP-Sialic acid synthase (CMAS). Genetic inactivation of the *Cmas* gene in the murine TSC line TS-Rs26 was achieved by applying the CRISPR/Cas9 technology (Fig. 1A). The guide RNA was designed to target the active site of the *Cmas* gene (Fig. S1A, B). Clonal lineages were established and clones with frame-shifts in both alleles were viable and termed *Cmas*^*−/−*^ (Fig. S1B, C). Loss of CMAS expression in *Cmas*^*−/−*^ TSC lines was confirmed by Western Blot analysis (Fig. 1B). Depletion of CMAS in murine embryonic stem cells has led to a complete loss of cell surface sialic acid [10]. To determine the sialylation status of control and *Cmas*^*−/−*^ TSC glycans, we used xCGE-LIF to analyze glycosphingolipid glycosylation. xCGE-LIF demonstrated that control TSC possessed two major (GD1a and GM1a) and three minor ganglioside species (GM2, disialyl-Lc4-Cer and sialyl-Lc4-Cer) (Fig. 1C). *Cmas*^*−/−*^ TSC however entirely lacked all ganglioside species, but exhibited markedly elevated levels of the neutral glycosphingolipids lactosylceramide (Lac-Cer), globotriaosylceramide (Gb3-Cer), gangliotriaosylceramide (Gn3-Cer), globotetraosylceramide (Gb4-Cer) and lactotetrasylceramide (Lc4-Cer) (Figs. 1C and S2A). Enzymatic treatment of glycosphingolipid derived glycans with Neu from *Arthrobacter ureafaciens* revealed that only control cells possess Neu sensitive glycolipid derived glycans (Fig. S2B, C). *Cmas*^*−/−*^ TSC globally lack sialylated structures on glycosphingolipids and mutant cells are thus further called “asialo”. Evaluation of different characteristic TSC markers by immunofluorescence and gene expression did not reveal major differences, demonstrating that loss of CMAS did not alter the stem cell character of TSC (Figs. 1D and S1D). We subsequently examined whether loss of Sia has an impact on the differentiation capacity of TSC by quantifying the expression of the differentiation markers *Prl3d1* and *Tpbpa* after 7 days of differentiation. qPCR analyses revealed no differences between control and *Cmas*^*−/−*^ TSC upon differentiation in medium with heat-inactivated serum (Fig. 1E).

### Asialo trophoblast stem cells are sensitive to complement activation

**Fig. 2:**
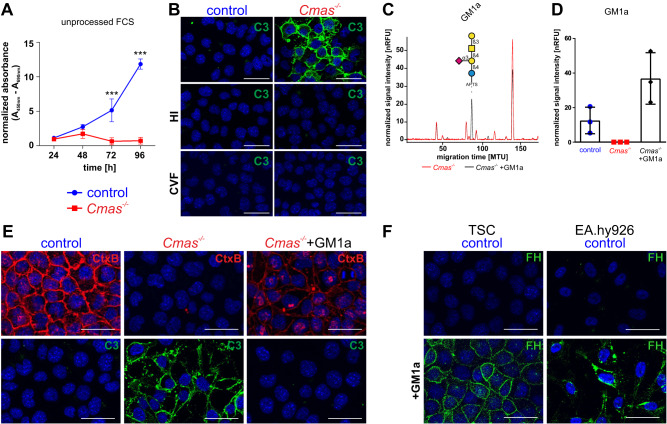
GM1a rescues complement sensitivity of *Cmas*^*−/−*^ TSC. **A** WST-1 proliferation assay of control and *Cmas*^*−/−*^ TSC in differentiation medium with unprocessed FCS. Absorbance was measured every 24 h after seeding for 4 days in three independent experiments. For statistical analysis a two-way ANOVA with Bonferroni’s multiple comparison post test was performed ***p < 0.001. Mean values are depicted with standard deviation. **B** Detection of C3 deposition by indirect immunofluorescence analysis of control and *Cmas*^*−/−*^ TSC in medium with untreated, heat inactivated (HI-FCS) or CVF treated (CVF-FCS) FCS for 3 h. **C** Overlay of xCGE-LIF electropherograms of APTS-labeled GSL-derived glycans of *Cmas*^*−/−*^ TSC with and without exogenously administered GM1a. Glycan notation for GM1a refers to the glycosphingolipid-derived glycan. MTU migration time unit, *n* = 3. Symbol nomenclature according to [31]. **D** Quantification of GM1a in control and *Cmas*^*−/−*^ TSC with and without exogenously administered GM1a, *n* = 3. Mean values are depicted with standard deviation. **E** Detection of endogenous and incorporated GM1a with CtxB in control TSC, *Cmas*^*−/−*^ TSC and *Cmas*^*−/−*^ TSC treated with GM1a in medium with untreated FCS. Detection of C3 deposition by indirect immunofluorescence analysis of control, *Cmas*^*−/−*^ TSC and *Cmas*^*−/−*^ TSC treated with GM1a in medium with untreated FCS, *n* = 3. **F** Detection of human complement FH by indirect immunofluorescence analysis of control TSC and EA.hy926 with and without prior incubation with GM1a in medium with unprocessed FCS. All nuclei were stained with DAPI and are shown in blue. All Scale bars = 50 μm.

When exposed to an intact complement system, *Cmas*^*−/−*^ trophoblasts show impaired differentiation in vivo [5]. Therefore, control and *Cmas*^*−/−*^ TSC were analyzed regarding their proliferation capacities by cultivation in differentiation medium with untreated serum, i.e., without heat-inactivation and intact complement system. In a WST-1 cell proliferation assay, control TSC showed increasing proliferation over the whole differentiation time-period of 96 h, while *Cmas*^*−/−*^ TSC revealed a marked reduction in proliferation (Fig. 2A). To analyze whether differentiating *Cmas*^*−/−*^ TSC also activate the complement system in vitro, we examined the presence of the central complement component C3 by indirect immunofluorescence. While control TSC showed no C3 reactivity, *Cmas*^*−/−*^ TSC revealed markedly increased deposition of C3 on the cell surface (Fig. 2B), which was abolished when heat-inactivated or cobra venom factor (CVF) treated serum was used (Fig. 2B). These results clearly demonstrate that loss of *Cmas*^*−/−*^ TSC viability is accompanied by activation of the complement system.

To verify that the deletion of *Cmas* was the primary reason for the observed complement sensitivity and to exclude off target effects, *Cmas*^*−/−*^ TSC were transfected with murine *Cmas*. Complementation of *Cmas*^*−/−*^ TSC not only restored sialylation (Fig. S3A, B), but also viability and differentiation in complement-active medium, demonstrating that loss of CMAS and subsequent loss of cell surface sialylation was responsible for the demise of *Cmas*^*−/−*^ TSC during in vitro differentiation (Fig. S3C).

Next, we analyzed whether xenogenic effects of complement-active FCS lead to the observed complement activation on murine TSC. We exposed control and *Cmas*^*−/−*^ TSC to either bovine or murine complement active serum under differentiation conditions. As before, *Cmas*^*−/−*^ TSC revealed a massively reduced viability in complement active bovine serum, while complement active murine serum supported growth of *Cmas*^*−/−*^ TSC under differentiation conditions (Fig. S4A). Since immunoglobulins (Ig) are a known activator of complement, especially in xenogenic conditions [11], we examined whether bovine Ig recognize murine cell surface components. Immunofluorescence analysis of cell surface bound bovine Ig revealed that *Cmas*^*−/−*^ TSC cultivated in complement active FCS indeed show reactivity for bovine Ig (Fig. S4B). To further elucidate if bovine Ig trigger complement activation on *Cmas*^*−/−*^ TSC we treated complement active FCS with protein G coupled beads, generating complement active FCS depleted of bovine Ig. While untreated FCS again did not support *Cmas*^*−/−*^ TSC growth, heat-inactivated, as well as protein G treated FCS enabled proliferation of *Cmas*^*−/−*^ TSC under differentiation conditions (Fig. S4C). These data suggest that murine TSC are able to regulate bovine complement proteins, while bovine Ig bind to murine TSC and induce a xenogenic complement attack, which cannot be regulated in the absence of cell surface sialylation. While the FCS driven xenogenic complement attack against murine TSC might not be physiologic, *Cmas*^*−/−*^ TSC represent a valuable tool to analyze complement inhibitors and routes of complement activation in the absence of sialylation.

### GM1a rescues regulation of complement activation on asialo trophoblast stem cells

The prominent occurrence of GM1a and GD1a in control TSC led us to the question whether these gangliosides directly contributed to the observed differences in complement regulation and thus viability. Although previous studies showed that GM1a exerts complement regulatory functions in artificial liposomes [12], other data suggests that ganglioside incorporation might even increase the susceptibility for complement mediated lysis [13]. To clarify this issue, we assessed whether GM1a is able to regulate complement activation in *Cmas*^*−/−*^ TSC. GM1a was administered to *Cmas*^*−/−*^ TSC cultures for 3 h and successful GM1a incorporation into the plasma membrane was controlled by xCGE-LIF (Fig. 2C). Quantification revealed that GM1a levels in treated *Cmas*^*−/−*^ TSC slightly exceeded control levels (Fig. 2D). Cell surface localization of the exogenously added GM1a was controlled using the GM1a specific receptor Cholera toxin subunit B (CtxB) (Fig. 2E). Control TSC showed strong CtxB reactivity, which was completely abolished in *Cmas*^*−/−*^ TSC, confirming our results from the xCGE-LIF analysis. CtxB reactivity in *Cmas*^*−/−*^ TSC could be restored upon treatment with GM1a, demonstrating that TSC are generally capable of incorporating GM1a into their plasma membrane (Fig. 2E). Analysis of C3 deposition revealed that GM1a treatment markedly decreased the C3 positive population of *Cmas*^*−/−*^ TSC when exposed to active complement in standard differentiation medium (Fig. 2E). This data suggest that incorporation of externally added GM1a improved the ability of *Cmas*^*−/−*^ TSC to regulate complement activation on the cell surface.

Since FH plays a crucial role in complement regulation and sialylated glycoconjugates are known to interact with FH [14], we assessed recruitment of purified human FH to the cell surface with and without prior administration of GM1a. FH was not detectable on untreated TSC and the human endothelial cell line EA.hy926 cells, whereas incorporation of exogenous GM1a increased binding of FH on both cell lines (Fig. 2F). This indicates that exogenously administered and incorporated GM1a represents a recognition motif for complement FH on murine TSC and EA.hy926 cells and suggests that FH may contribute to the complement regulatory function of GM1a.

### GM1a exerts complement regulatory functions on erythrocytes and endothelial cells

**Fig. 3:**
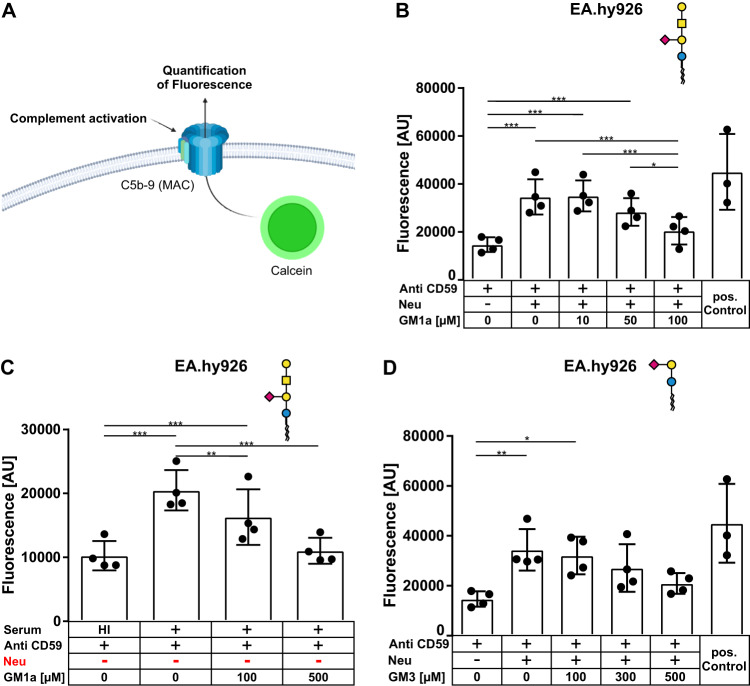
Exogenously administered GM1a protects EA.hy926 from complement attack. **A** Schematic representation of the experimental setup. Fluorescent calcein can be measured in the supernatant only after complement mediated formation of C5b-9. Created with biorender.com. **B** GM1a improves complement regulation on the endothelial cell line EA.hy926. A Calcein release assay was used to determine the complement activation on the human endothelial cell line EA.hy926. Cells were desialylated with Neu and subsequently incubated with different concentrations of GM1a. After treatment with human serum, the supernatants were measured for fluorescence at 494 nm. Shown are absolute fluorescent values and mean values ± SD. All cells were treated with anti-CD59 to block the inhibition of C5b-9 formation. For the positive control, cells were lysed with detergents to release the entire Calcein. **C** GM1a improves complement regulation on the endothelial cell line EA.hy926 also without prior removal of sialic acid. The same calcein release assay as in (**A**) was performed but without neuraminidase treatment. For statistical analysis a repeated measures ANOVA with Bonferroni’s multiple comparison post test was performed *p < 0.05, **p < 0.01, ***p < 0.001. Mean values are depicted with standard deviation. **D** GM3 improves complement regulation on the endothelial cell line EA.hy926. A Calcein release assay was used to determine the complement activation on the human endothelial cell line EA.hy926. The same calcein release assay as in (**A**) was performed but with GM3 instead of GM1a treatment.

The complement regulatory properties of GM1a in *Cmas*^*−/−*^ TSC raised the question if this feature of GM1a was applicable to other complement sensitive biological systems. A cell type that is prone for complement-mediated injury is the endothelium, which in vivo is permanently exposed to complement proteins. To examine whether GM1a is also capable of regulating complement mediated injury on endothelial cells we used EA.hy926 cells in a calcein release assay that was previously used to study complement activation on endothelial cells (Fig. 3A) [15]. Control cells without further treatment showed a mean fluorescent signal of 14,702 ± 3069 AU in the cell culture supernatant after exposure to complement active serum (Fig. 3B). Cells that were additionally treated with Neu had a significantly higher fluorescent signal of 34,622 ± 7378 AU, indicating increased calcein release due to C5b-9 complex formation as a result of complement activation. Additional exposure with 100 µM GM1a abolished this difference. Furthermore, treatment with 100 µM GM1a also showed significantly decreased signals compared to untreated or 10 µM GM1a treated cells. These data show that exogenously administered GM1a regulated complement activation on complement sensitized endothelial cells. Next, we analyzed whether the complement regulatory function of GM1a also holds true for regularly sialylated endothelial cells. Treatment of EA.hy926 cells with 500 µM GM1a decreased fluorescence signals in the calcein release assay further to a level comparable to cells exposed to heat-inactivated serum (Fig. 3C). This indicates that the complement regulatory function of incorporated GM1a is not restricted to cells with impaired sialylation. Next, we asked if also other sialylated glycosphingolipids containing the sialyllactose motif are able to improve complement regulation. Therefore, we used the same experimental setup with EA.hy926 cells with GM3, which is similar to GM1a but lacks the GalNAc-Gal elongation of the sialyllactose-ceramide. Treatment of EA.hy926 cells with GM3 reduced complement mediated fluorescence in the supernatant from 34,415 ± 8291 AU to 20,942 ± 4184 AU, but without reaching statistical significance (Fig. 3D).

**Fig. 4:**
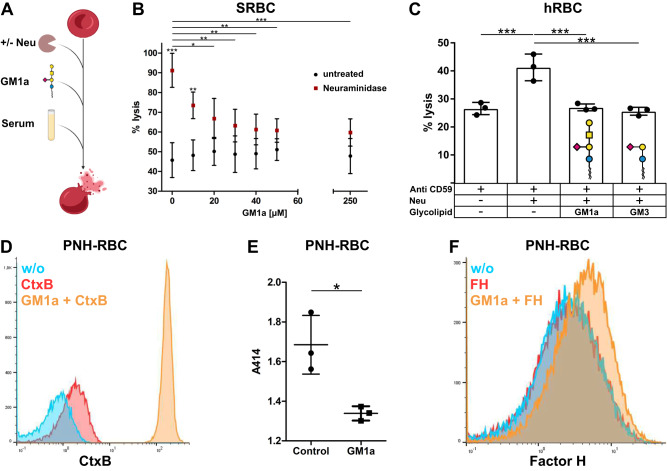
GM1a protects healthy and PNH-RBC from human complement. **A** Schematic representation of the experimental setup. Free hemoglobin can be measured colorimetrically as a surrogate for complement mediated hemolysis of sheep or human RBC. Created with biorender.com. **B** SRBC were left untreated or sensitized for activation of the alternative complement pathway by incubation with Neu. Furthermore, control and Neu treated SRBC were incubated with increasing concentrations of GM1a. Hemolysis was determined at 415 nm after incubation of SRBC with 16.6% human complement active serum in DGHB-Mg-EGTA. Hemolysis is plotted relative to SRBC incubated with only red cell lysis buffer, which was defined as 100% lysis. *n* = 3. **C** Control, Neu sensitized or Neu sensitized and subsequently GM1a or GM3 (both 50 µM) treated human RBC were incubated with human complement active serum in DGHB-Mg-EGTA. Hemolysis was plotted relative to human RBC incubated with only red cell lysis buffer, which was defined as 100% lysis. All cells were treated with anti-CD59 to block the inhibition of C5b-9 formation. For statistical analysis a repeated measures ANOVA with Bonferroni’s multiple comparison post test was performed *p < 0.05, **p < 0.01, ***p < 0.001. Mean values are depicted with standard deviation. **D** Detection of endogenous and incorporated GM1a with CtxB in PNH-RBC and PNH-RBC treated with GM1a. **E** PNH-RBC and PNH-RBC with subsequent GM1a (100 µM) treatment were incubated with human complement active serum in DGHB-Mg-EGTA. Absorbance of free hemoglobin due to hemolysis was measured at 414 nm. **F** Detection of human FH by flow cytometry analysis of PNH-RBC with (FH + GM1a) and without (FH) prior incubation with GM1a. Subsequently PNH-RBC were exposed to FH (100 µg/ml) and FH recruitment was detected by anti-FH via flow cytometry.

A frequently used cellular system to assess complement regulatory potential of novel therapeutic approaches are Neu treated sheep red blood cells (SRBC). SRBC are only sensitive for complement mediated lysis by human serum after the cell surface sialic acid is cleaved off by Neu. This long-standing read-out system for complement mediated hemolysis is an established model to test the effect of complement inhibitors on the alternative complement pathway (Fig. 4A) [16]. We could observe a dose-dependent decrease of SRBC lysis when Neu treated SRBC were pre-treated with GM1a, which confirms our previous observations with EA.hy926 cells (Fig. 4B). In the following, we analyzed whether GM1a is also capable of regulating human complement on sensitized human red blood cells (hRBC). hRBC were sensitized for complement-mediated lysis by a functional blockade of the complement regulatory protein CD59 with a monoclonal antibody and Neu treatment [15]. Control hRBC showed lysis of about 27% that increased to 41% after Neu treatment. Administration of GM1a to Neu-treated hRBC prior to incubation with complement active serum decreased lysis to 27% (Fig. 4C). Similar to GM1a, also GM3 significantly reduced complement mediated lysis of hRBC (Fig. 4C). While Neu treatment significantly sensitized hRBC to complement mediated lysis, GM1a and GM3 treatment was able to reduce lysis of Neu-treated hRBC back to control levels. Summarized, exogenously applied GM1a and GM3 exerted complement regulatory functions on hRBC.

Next, we asked whether GM1a is able to regulate complement activation under pathophysiological conditions. Therefore, we used red blood cells from a patient with PNH in a hemolysis assay. Patients with PNH have a subset of hematopoietic stem cells that lack Glycosylphosphatidylinositol (GPI)–anchored proteins, such as the complement regulators CD55 and CD59. RBC originating from these GPI-negative stem cells are prone for complement-mediated hemolysis. As before, we used CtxB to assess the incorporation of GM1a into PNH-RBC. While the RBC population of patients with PNH is divided into GPI+ and GPI− cells, pretreatment of PNH-RBC with GM1a led to a homogenous population of CtxB positive cells (Figs. 4D and S5). This suggests that GPI+ and GPI− RBC incorporate GM1a to a similar extend. Exposure of PNH-RBC to complement active human serum led to hemolysis in the untreated sample, which was significantly reduced if the PNH-RBC were pretreated with exogenous GM1a (Fig. 4E). In the following, we analyzed if GM1a also increases the recruitment of FH to PNH-RBC. Similar to TSC and EA.hy926 cells, exogenous GM1a promoted the recruitment of FH to the cell surface of PNH-RBC and thus likely mediates the complement regulation observed in GM1a treated PNH-RBC (Fig. 4F).

## Discussion

The complement system requires tight regulation in order not to compromise host cell viability. Therefore, it is not surprising that numerous pathologies include dysregulation of the complement pathway (e.g., PNH, C3 glomerulopathy, atypical hemolytic uremic syndrome (aHUS) and ischemia reperfusion injury) [1]. Most therapeutic approaches address exuberant complement activity by inhibition of C3 or C5 convertases, e.g., AMY-101 (Amyndas) and the monoclonal antibody eculizumab, respectively. While these strategies effectively inhibit full-blown complement activation, they also systemically inhibit complement activation at the risk of infections with encapsulated bacteria and do not address the underlying deficiencies in complement defense on the host cell surface [17, 18]. Restoration of complement regulation directly on the cell surface would require complement defense molecules that are able to integrate into the plasma membrane of affected host cells. A proof that this concept is generally applicable is the membrane-targeted complement inhibitor Mirococept (APT070), which has been successfully employed in in vivo studies, including improved graft survival of cold stored donor organs in a rat transplantation model [19].

Here, we describe the discovery of a novel function of the monosialoganglioside GM1a as a membrane targeted complement regulatory molecule. GM1a showed complement inhibiting potential as an endogenously present molecule or after integration into the cell surface when exogenously added. Previous studies attributed to ganglioside treatment of hRBC an increase in complement sensitivity [13], while GM1a incorporation into artificial liposomes reduced complement activation [12]. The complement regulatory potential of GM1a observed in our study significantly expands these earlier observations and clearly demonstrates that GM1a integrates into the plasma membrane of hRBC, endothelial and placental cells and negatively regulates complement activation. Most importantly, the complement regulatory potential of GM1a was not restricted to cells with impaired sialylation, but actually improved complement mediated hemolysis of PNH-RBC under pathophysiological conditions. This complement regulatory potential of GM1a is likely mediated by increased recruitment of the complement regulatory protein FH to the cell surface of GM1a treated cells. FH consists of 20 complement-control protein (CCP) domains and is *inter alia* recruited to the cell surface via C3b binding sites; therefore, it preferably binds to cell surfaces with active complement events. Moreover, FH possesses a Sia binding pocket that accommodates cell surface glycans with a terminal sialic acid in α-2,3 linkage and STD NMR analyses showed that FH interacts with the glycan portion of GM1a and GM3 [9, 20]. While recruitment of FH could readily explain the complement regulatory potential of exogenous GM1a, it is not clear why FH recruitment was not detected in control TSC, which according to our analyses contain and display endogenous GM1a. One explanation might be that GM1a treatment raised GM1a concentrations beyond endogenous levels. In addition, due to its short spatial extent GM1a might not be readily available as FH ligand. Especially in contrast to bulky cell surface glycoproteins that could easily mask endogenous GM1a and thus block FH-GM1a interactions. Treatment with GM1a micelles however, could result in the formation of relatively large GM1a patches on the cell surface, which might be more accessible for fluid phase proteins. Additionally, it was shown that exogenous administration of GM1a induces changes in the organization of the plasma membrane, which could make GM1a and other FH receptors more readily accessible [21]. While we still lack a precise understanding of how different cell surface glycans contribute to the interaction of FH with the cell surface, our data show that glycomic approaches like xCGE-LIF offer a great opportunity to identify glycans with such potential in future studies. Moreover, we could show that also GM3, which is comprised of a sialyllactose bound to ceramide, is able to improve complement regulation. This suggests that all gangliosides that contain a mono-sialyllactose motif, which is a known FH binding ligand [9], might improve complement regulation when exogenously added.

Our data emphasize once more the complement sensitive nature of trophoblast cells and provide first evidence that exogenously administered GM1a has complement regulatory functions in important cellular systems of the hematological compartment. GM1a is an intensely studied ganglioside that is produced naturally in the human body. It accounts for about 28% of the total human brain gangliosides and is currently being studied in clinical trials in the treatment of neurological diseases because of its neuroprotective function [22–24]. Importantly, these clinical trials showed no adverse immuno-stimulant effects after injection of GM1a, suggesting that GM1a treatment as therapeutic intervention in complement related diseases would be similarly safe [22, 23]. Taken together our findings highlight the potential of GM1a, and possibly other gangliosides, as new therapeutic agents for membrane-targeted complement inhibition which might also be of interest for many different pathologies, including neurodegenerative, hemolytic, renal and autoimmune diseases [1, 25–27].

## Methods

### Stem cell culture

The TS-Rs26 cell line, originally from the Rossant Lab (Toronto, Canada), was a kind gift of the Hemberger lab, University of Calgary, Canada. Standard culture conditions are described in Supplementary Methods. Transfection was performed using Lipofectamine (Thermo Fisher) according to the manufacturer’s protocol. Depletion of C3 from fetal bovine serum was performed using 0.83 µg/ml CVF (Quidel) for 30 min at 37 °C. Neu treatment of cells was conducted with 0.13 mg/ml purified *Arthrobacter ureafaciens* neuraminidase (in house production according to [28]) in serum-free TSC medium for 30 min at 37 °C.

### Generation of *Cmas*^*−/−*^ TSC

CRISPR-Cas9-mediated gene ablation was carried out using a guide RNA targeting *Cmas* exon 4 (5’-TGTCGACGAGGCCGTTTCGC-3’) in the plasmid pX330-U6-Chimeric_BB-CBh-hSpCas9 (Addgene plasmid #42230 from Feng Zhang) as used before [29]. TSC were cotransfected with GFP-expressing plasmid peGFP-CI (Clontech) and sorted for GFP-positive cells in a 96-well plate at the central research facility cell sorting at MHH. Control TSC were mock transfected and sorted. Screening for *Cmas*^*−/−*^ TSC was performed using primer targeting exon 4 (5’-GTCTGCATTCTGAGGGGAGT-3’ and 5’- AGAGCACAACACAGAAGGCT-3’) followed by Sanger sequencing (Eurofins).

### xCGE-LIF

As described previously, glycosphingolipid (GSL) glycosylation was assessed by multiplexed capillary gel electrophoresis coupled to laser induced fluorescence (xCGE-LIF) detection using an ABI PRISM® 3100-Avant Genetic Analyzer (advanced biolab service GmbH, Munich, Germany) [30]. The procedure is described more in detail in Supplementary Methods. Annotation of peaks was performed by migration time alignment to our in-house database [30].

### qRT-PCR

RNA preparation with TRIzol® (invitrogen) and cDNA synthesis were performed according to the manufacturer’s protocols. Sequences of qPCR Primer (Sigma-Aldrich) are listed in Table S1. qPCRBIO SyGreen Lo-ROX Mastermix (Nippon Genetics Europe, Germany) was used on a QuantStudio 3 thermocycler. Ct values were normalized to three reference genes (Pgk1, Sdha, Ywhaz). Three biological replicates for each independent clone were used for all qPCR data.

### Immunofluorescence staining

For staining of cultured cells, TSC were seeded on glass coverslips. Fixation was performed in 4% PFA/PBS and when needed permeabilised using 0.2% Triton X-100 for 10 min. Blocking and antibody incubations were carried out in 1% BSA/PBS for 1 h at RT. Detailed dilutions of used antibodies are listed in Table S2. Processed coverslips were mounted in Vectashield mounting medium (Vectorlabs, Burlingame, CA, USA), containing DAPI as counter-stain for nuclei. Images were taken with Zeiss Observer.Z1 microscope equipped with an AxioCam MRm digital camera and ApoTome module. Analysis was performed using Zeiss Zen software.

### Proliferation assay

TSC were seeded in 96-well-plates at a density of 300 cells/well and incubated under differentiation conditions. Proliferation of differentiating TSC was analyzed using WST-1 reagent (Roche, Basel, Switzerland) 24, 48, 72 and 92 h after seeding. Ten µl WST-1 reagent was added to the well and incubated for 4 h at 37 °C. Measurement of absorbance at 450 and 690 nm as reference wavelength determined cell proliferation. This assay was performed three times with technical triplicates.

### GM1a incorporation of TSC

Prior to the incorporation of GM1a into the cell membrane, TSC were seeded in a 12-well plate and incubated at stem cell conditions. After 72 h, TSC were incubated for 3 h with serum-free TSC medium and a concentration of 800 µM GM1a (Carbosynth Biosynth, United Kingdom) at 37 °C. Subsequently TSC were either directly fixed as described earlier or passaged to a new 12-well plate for differentiation.

### Complement FH binding assay

TSC were seeded in a 12-well plate and incubated at stem cell conditions for ~72 h. GM1a incorporation was performed in selected wells as described above. Afterwards, TSC were washed two times with serum-free TSC medium. Incubation with human complement FH (Complement Technology, Texas, USA) was carried out at 37 °C for 30 min in serum-free TSC medium at a final concentration of 10 µg/ml. Subsequently, TSC were directly fixed as described above and used for indirect immunofluorescence analysis. For FH binding to PNH-RBC, 50 µl of 5*10^7^ erythrocytes/ml were treated with our without 100 µM GM1a for 60 min at 37 °C and subsequently incubated with 100 µg/ml FH for 30 min at 37 °C. FH binding was assessed via flow cytometry using goat anti-FH and anti-goat Alexa 647 (Invitrogen).

### Western blotting

Protein lysates were prepared in RIPA buffer and BCA Assay (Thermo Scientific) was used to determine protein concentration. Equal protein amounts were separated in a 12% SDS-PAGE and blotted onto PVDF membrane. Primary antibody incubation was performed over night at 4 °C with subsequent secondary antibody incubation for 1 h at RT. Detailed dilutions of used antibodies are listed in Table S2. Enhanced chemiluminescence was utilized for detection.

### EA.hy926 cell culture

EA.hy926 cells were grown in DMEM high glucose (biowest) with 10% heat-inactivated FCS (Merck) with 1% penicillin/streptomycin (PAN-biotech) at 37 °C in humidified incubators containing 5% CO_2_. Passaging of subconfluent cells was carried out using TrypLE (gibco). Medium was changed every other day.

### PNH cells

Use of human blood samples was approved by the institutional review board/ethics committee. The patient provided written informed consent. Peripheral blood was drawn from a patient with PNH in stable condition during therapy with ravulizumab and anticoagulated with 1/10 vol. 3.8% sodium citrate. Red blood cells were isolated and washed in PBS at least two times.

### Hemolysis assay

The hemolysis assays using human and sheep red blood cells are described in detail in the Supplementary Methods.

### Calcein release assay

The assay was performed as described before [15]. Description of the procedure can be found in Supplementary Methods.

### Statistical analysis

Statistical analysis was performed using GraphPad Prism 7 software (GraphPad, San Diego, CA, USA). One-way ANOVA and mixed two-way ANOVA followed by Bonferroni’s post hoc test were applied as indicated. Normality and equality of variances were assessed by the Shapiro–Wilk and the Brown–Forsythe test, respectively. *p < 0.05, **p < 0.01, ***p < 0.001.

### Supplementary information

Original Data File

Supplemental Material

## Data Availability

The datasets generated during and/or analyzed during the current study are available from the corresponding authors on reasonable request.
