# Supplementary material for: The monosialoganglioside GM1a protects against complement attack
Source: Cell Death Discov. 2023 Oct 25;9:395. doi: 10.1038/s41420-023-01686-6 (PMC10600102; doi:10.1038/s41420-023-01686-6)

Full uncropped gels

Figure 1, B

Anti CMAS Western Blot

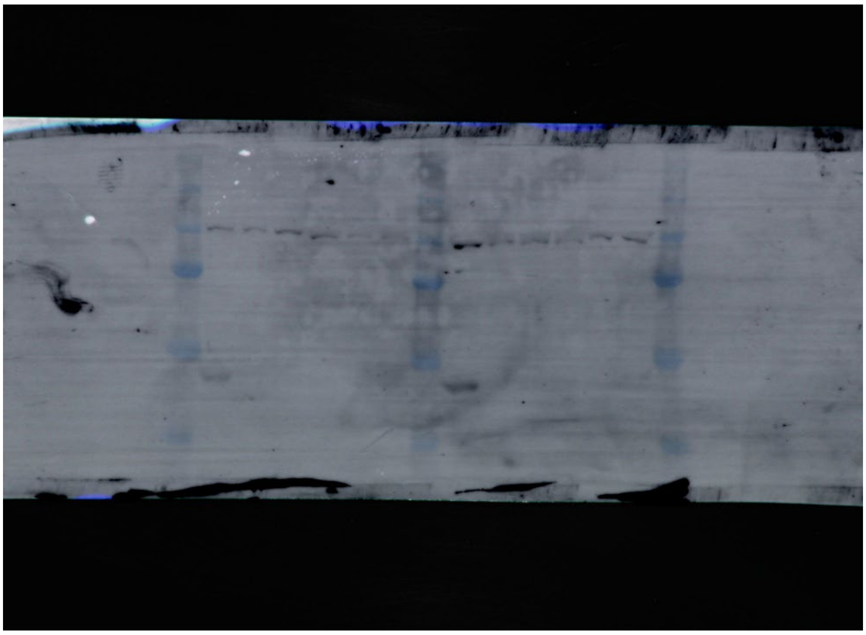

Anti Actin

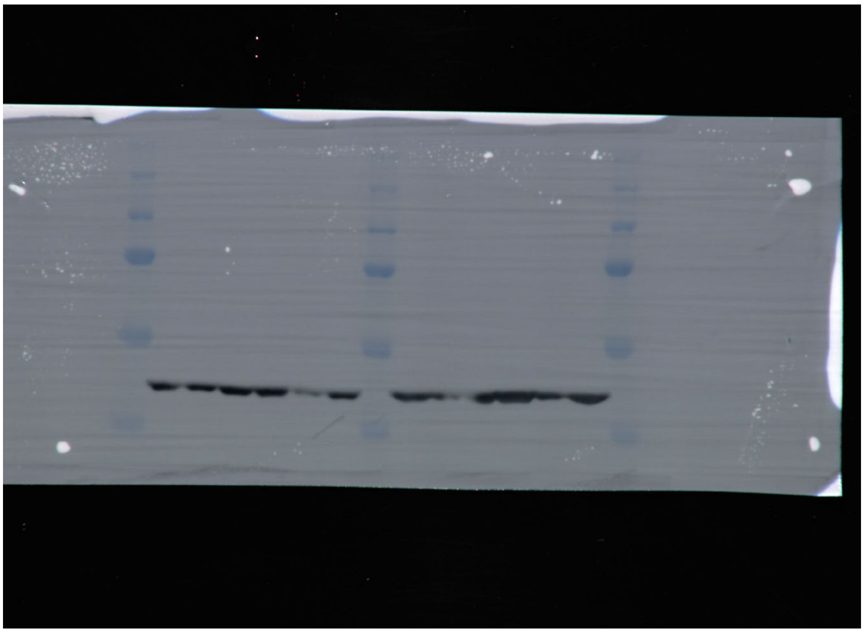

Figure S3, A

Anti Flag

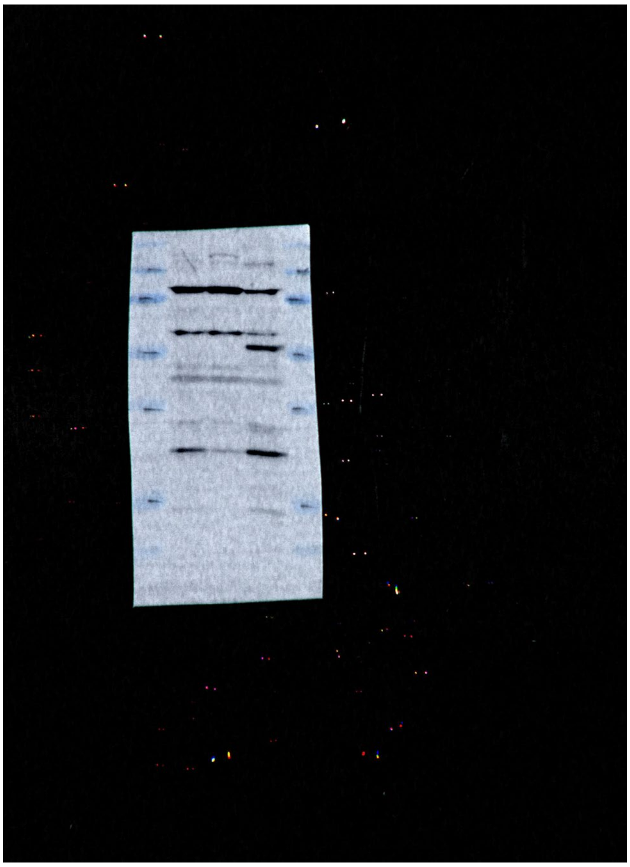

Anti Actin **Anti CMAS**

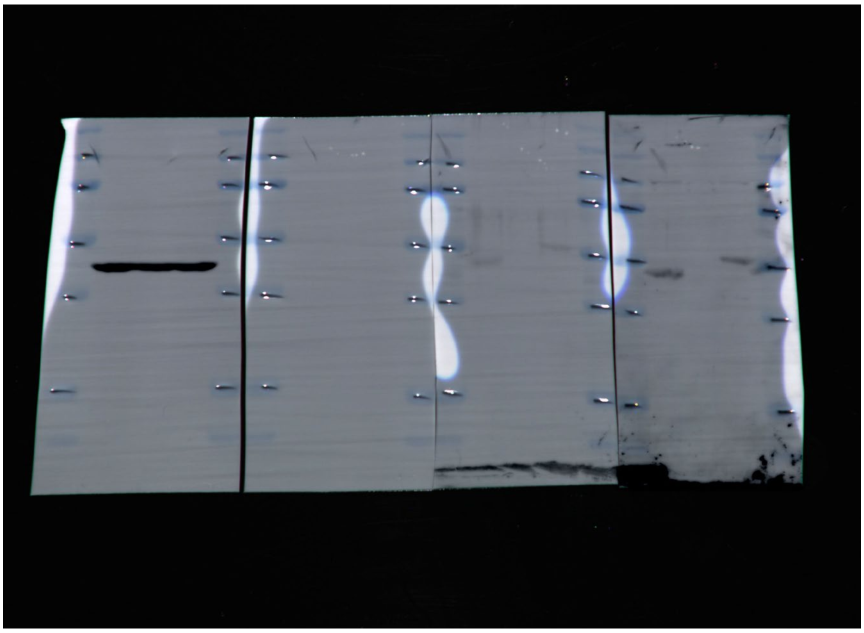

Supplement: Supplementary file 1 — Original Data File [file 41420_2023_1686_MOESM1_ESM.pdf]
