# Supplementary material for: The monosialoganglioside GM1a protects against complement attack
Source: Cell Death Discov. 2023 Oct 25;9:395. doi: 10.1038/s41420-023-01686-6 (PMC10600102; doi:10.1038/s41420-023-01686-6)

**Supplemental Methods**

**Stem Cell Culture.** Basal TSC medium consisted of RPMI 1640 medium (biowest) with 1 % penicillin/streptomycin (PAN-biotech), 1mM sodium pyruvate (Merck), 50 µM 2-mercaptoethanol (Sigma-Aldrich) and 20 % fetal bovine serum (Merck). For stem cell culture, fetal bovine serum was heat inactivated for 30 min at 56°C. Undifferentiated TSC were cultured with 70 % of the medium pre-conditioned on irradiated murine embryonic fibroblasts (Merck) supplemented directly before use with 1 µg/ml heparin (Sigma-Aldrich) and 25 ng/ml FGF4 (Reliatech) at 37°C in humidified incubators containing 5 % $CO_2$. Passaging of subconfluent cells was carried out using TrypLE (gibco). Medium was changed every other day. For differentiation experiments, standard differentiation medium (TSC medium without FGF4, Heparin and MEF conditioned medium) was used. In differentiation experiments with murine complement active serum (Innovative research), standard differentiation medium was used without FCS, but with 20 % murine complement active serum. Bovine Ig depleted FCS was generated by incubation of FCS with Protein G beads (Amersham, UK) over night at 4°C with gentle agitation.

**xCGE-LIF.** For GSL analysis, glycan head groups of previously extracted glycosphingolipids were released by LudgerZyme Ceramide Glycanase (Ludger, Oxfordshire, UK), fluorescently labelled with 8-Aminopyrene-1,3,6-trisulfonic acid trisodium salt (APTS), and subjected to xCGE-LIF. For inter-sample quantitative comparison of signal intensities, a defined amount of APTS-labelled Glyko® Oligomannose 6 (Man6, Prozyme, Hayward, CA) was spiked into each sample. The obtained Man6 signal intensity was set to 1 normalized signal intensity (nRFU) and was used for normalization of peak intensities.

**Hemolysis assay.** Human whole blood was obtained by venipuncture and erythrocytes were isolated by centrifugation for 5 min at 1.000 x g. RBCs were washed two times in PBS and stored in Alsever's solution at 4°C with light agitation. Desialylation was performed under continuous agitation with 0.13 mg/ml purified *Arthrobacter ureafaciens* neuraminidase (AU 54) (1) for 30 min at 37°C in PBS. Simultaneously, sensitising of erythrocytes was achieved by incubation with anti-CD59 antibody (2 µg/ml). For the incorporation of GM1a or GM3, erythrocytes were incubated for 1 h at 37°C in PBS in agitation with the respective concentrations. Erythrocytes were washed and resuspended in DGHB-Mg-EGTA (HEPES 4.2 mM, NaCl 59 mM, $MgCl_2$ 7 mM, EGTA 10 mM, Glucose 2.08 % (w/v), Gelatine 0.08 % (w/v)) (2) buffer to a final concentration of $5*10^7$ erythrocytes/ml. In a 96-well microtiter plate 50 µl erythrocytes were mixed with human serum diluted in DGHB Mg-EGTA to a final volume of 100 µl and incubated for 30 min at 37°C with continuous shaking. Subsequently, 150 µl NaCl 0.9 % were added

and the plate was centrifuged at 1000 xg for 5 min. Supernatant was transferred to a flat-bottomed 96-well plate and absorption at 414 nm was detected in a plate reader. All samples were measured in duplicates. Negative controls contain no serum. 100 % erythrocyte lysis was carried out by adding 200µl RBC lysis buffer ($NH_4Cl$ 155 mM, $NaHCO_3$ 12 mM, 0.1 mM EDTA) to 50 µl RBC. For hemolysis assays of PNH-RBC incubation with anti-CD59 was omitted and 100µM GM1a and complement active human serum serotype AB were used. Experiments with human samples were approved by the local ethics committee (9371_BO_S_2020). Hemolytic assay with sheep red blood cells (SRBC) (Innovative research, Novi, MI, USA) were performed as described for human RBC. However, inucubation with anti-CD59 was omitted.

**Calcein release assay.** In brief, EA.hy926 cells ($10^4$ per well) were treated for 30 minutes at 37°C with Neu, incubated for 30 minutes with Calcein AM (25 µM) and GM1a or GM3 (Avanti Polar Lipids, United States) of varying concentrations was added for 1h at 37°C. To induce a complement attack the cells were then incubated with 15 % human serum together with anti-CD59 (10 µg/ml). After 30 minutes at 37°C fluorescence of the supernatant at 494 nm was measured.

**CtxB flow cytometry.** To confirm GM1a incorcopration in PNH-RBC, 50µl of $5*10^7$ erythrocytes/ml were treated with our without 100 µM GM1a for 60 min at 37°C and subsequently incubated with CtxB-Alexa 488 (1:400). CtxB binding was assessed via flow cytometry.

**Supplemental Figures**

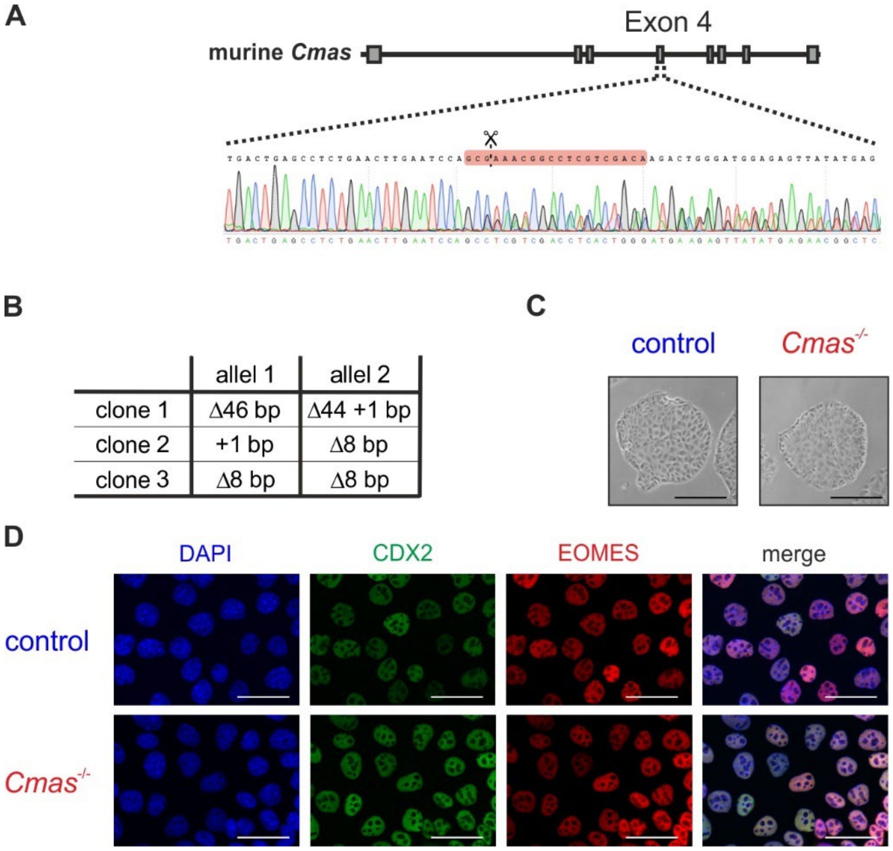


| | allel 1 | allel 2 |
|---|---|---|
| clone 1 | Δ46 bp | Δ44 +1 bp |
| clone 2 | +1 bp | Δ8 bp |
| clone 3 | Δ8 bp | Δ8 bp |

**Figure S1. Genetic deletion of *Cmas* does not alter the stem cell status of TSC.** (**A**) Scheme of the murine *Cmas* gene and sequencing data of one representative *Cmas*$^{-/-}$ clone with indels at the target site in exon 4. (**B**) The table depicts the size of indels in exon 4 of *Cmas*$^{-/-}$ clones determined by sequencing. (**C**) Brightfield images of control and one representative *Cmas*$^{-/-}$ TSC cultivated under standard conditions. Scale bars = 250 µm. (**D**) Detection of EOMES and CDX2 by indirect immunofluorescence analysis of control and one representative *Cmas*$^{-/-}$ TSC clone. Nuclei were stained with DAPI and are shown in blue. Scale bars = 50 µm.

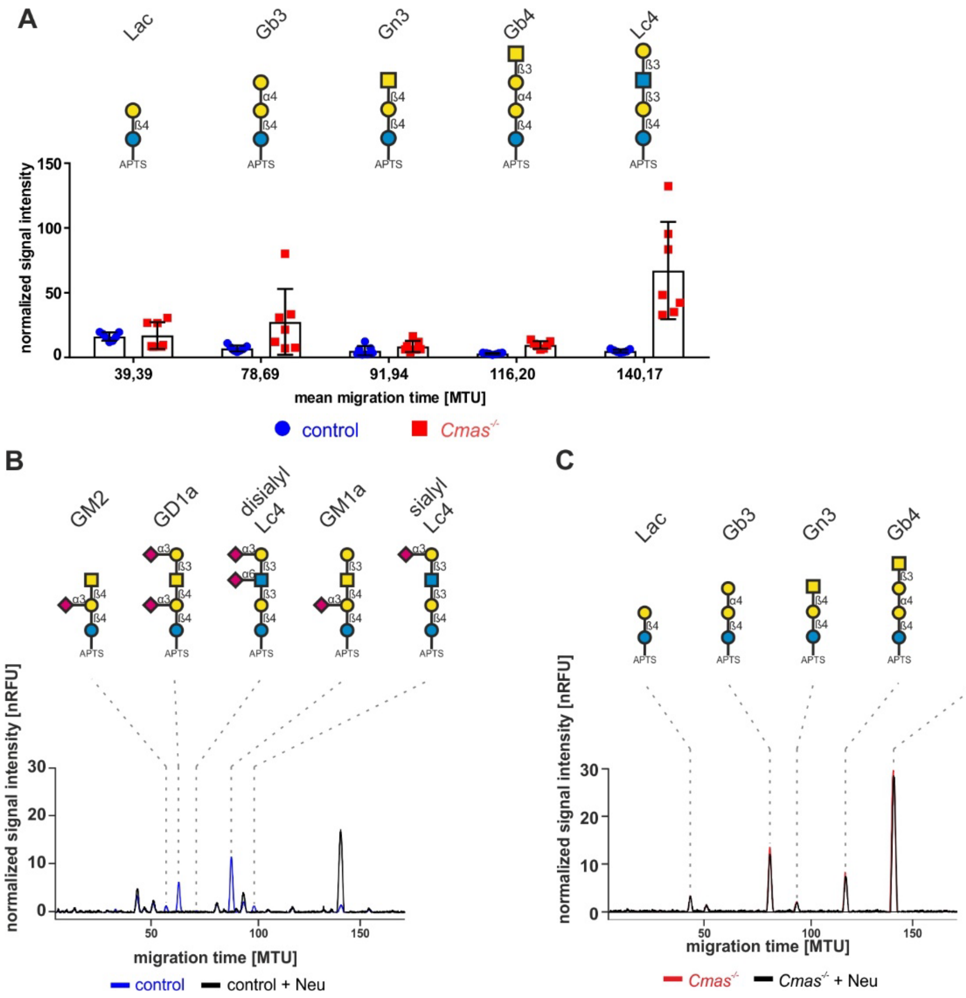


**Figure S2. *Cmas$^{-/-}$* TSC lack sialylated glycosphingolipids.** (**A**) Quantification of neutral glycans derived from glycosphingolipids of control and one representative *Cmas$^{-/-}$* TSC clone using xCGE-LIF. Glycan notations for GM2, GD1a and GM1a refer to the respective glycosphingolipid derived glycan. For inter-sample comparisons signal intensities were normalized to Man6 (nRFU). MTU: migration time unit. Symbol nomenclature according to [3]. n=7 Mean values are depicted with standard deviation. (**B-C**) xCGE-LIF analysis of glycans derived from glycosphingolipids. Glycan notations for GM2, GD1a and GM1a refer to the respective glycosphingolipid derived glycan. Overlay of xCGE-LIF electropherograms of APTS-labeled GSL derived glycans of control (**B**) and *Cmas$^{-/-}$* TSC (**C**) with or without prior treatment with Neu. For inter-sample comparisons signal intensities were normalized to Man6 (nRFU). MTU: migration time unit. Symbols nomenclature according to (3).

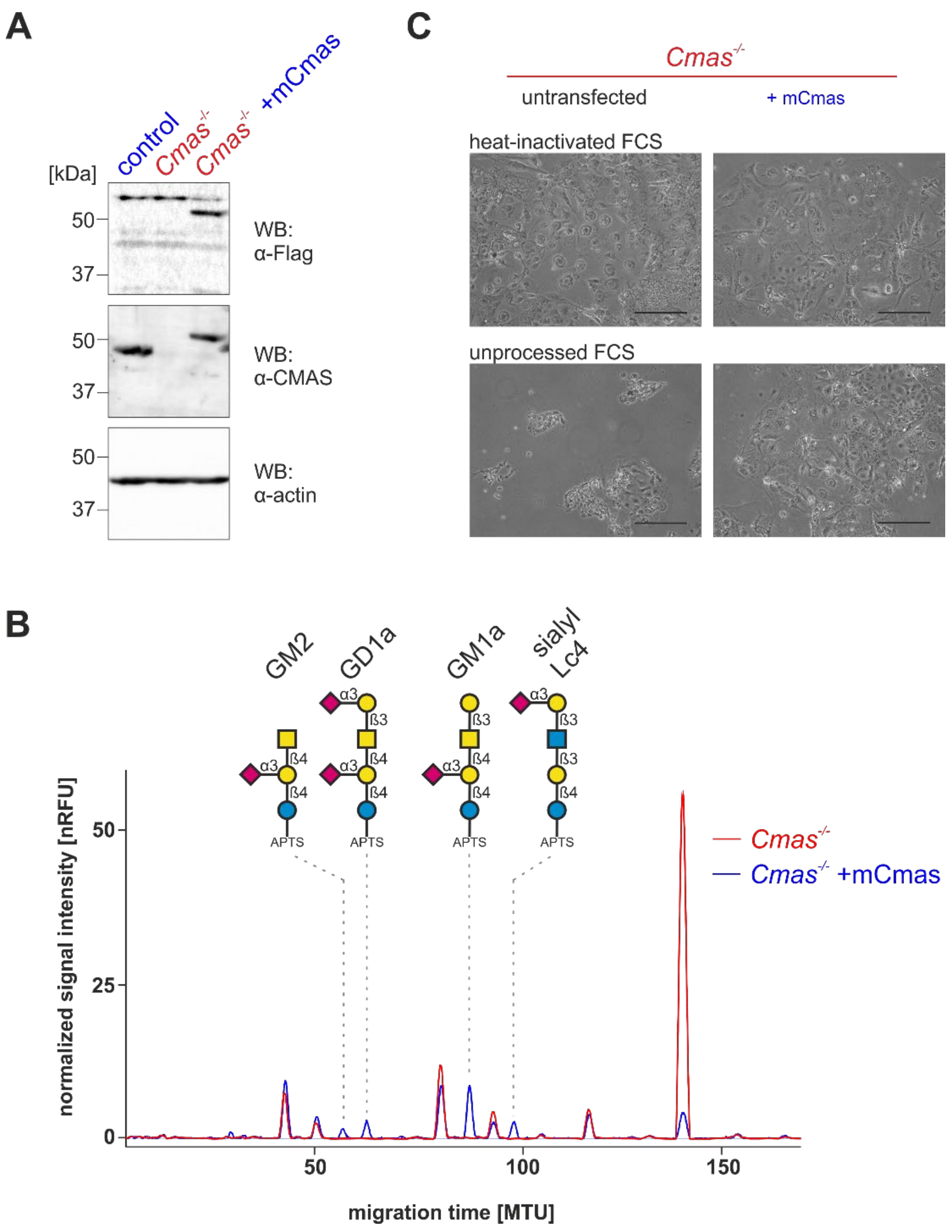


**Figure S3. Recomplementing of *Cmas-/-* TSC with murine Cmas rescues *Cmas-/-* TSC from complement attack.** (**A**) Control, *Cmas-/-* and recomplemented *Cmas-/-* TSC were lysed and separated by SDS-PAGE, blotted onto PVDF membrane and immunostained with anti-CMAS antibody and anti-Flag antibody. Anti-actin immunostaining was used as loading control. (**B**) Overlay of xCGE-LIF electropherograms of APTS-labeled GSL-derived glycans of *Cmas-/-* and recomplemented *Cmas-/-* TSC. Glycan notations for GM2, GD1a and GM1a refer to the respective glycosphingolipid derived glycan. For inter-sample comparisons, signal intensities were normalized to Man6 (nRFU). MTU: migration time unit, n=3. Symbol nomenclature according to (3). (**C**) Brightfield images of *Cmas-/-* and recomplemented *Cmas-/-* TSC after 7 days of differentiation in medium with unprocessed or heat-inactivated FCS. Scale bars = 250 µm.

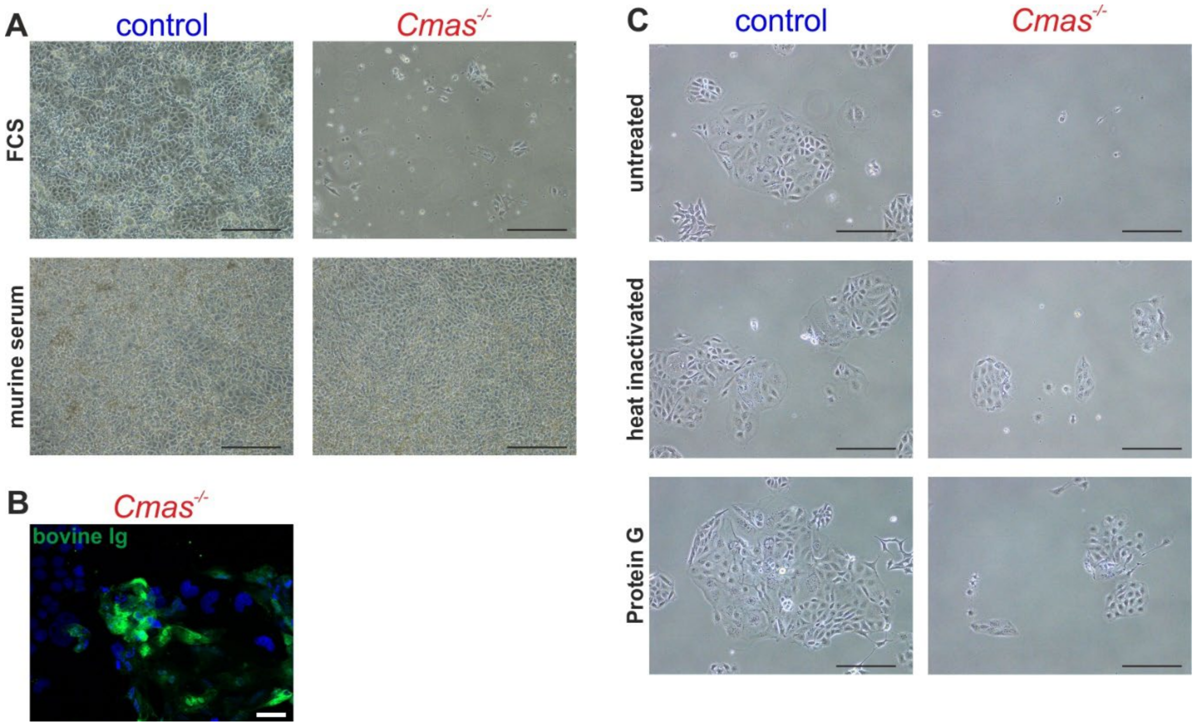


**Figure S4. FCS induced complement activation on *Cmas*$^{-/-}$ TSC is driven by bovine immunoglobulins.** (**A**) Brightfield images of control and *Cmas*$^{-/-}$ TSC cultured in differentiation medium supplemented either with complement active FCS or complement active murine serum. Scale bars = 250 µm. (**B**) Detection of bovine Ig (shown in green) by indirect immunofluorescence analysis of differentiated *Cmas*$^{-/-}$ TSC. Nuclei were stained with DAPI and are shown in blue. Scale bars = 50 µm.(**C**) Brightfield images of control and *Cmas*$^{-/-}$ TSC cultured for two days in differentiation medium supplemented either with untreated (active complement), heat-inactivated (inactive complement) or protein G treated (immunoglobulin depleted, active complement) FCS. Scale bars = 250 µm.

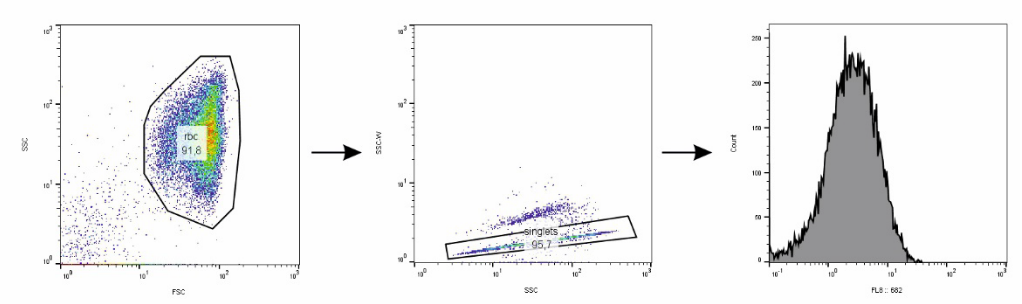


**Figure S5. Gating strategy for flow cytometry analysis.** Red blood cells were selected from a forward scatter-area vs sideward scatter-area dot plot. Single cells were subsequently selected in a sideward scatter-area vs sideward scatter width dot plot. This population was analyzed for FH/CtxB reactivity.

## Supplemental Tables

### Table S1: qPCR primer sequences

| | |
|---|---|
| *Elf5 fwd* | ATTCGCTCGCAAGGTTACTCC |
| *Elf5 rev* | GGATGCCACAGTTCTCTTCAGG |
| *Esrrb fwd* | AGTACAAGCGACGGCTGG |
| *Esrrb rev* | CCTAGTAGATTCGAGACGATCTTAGTCA |
| *Pgk1 fwd* | CTGACTTTGGACAAGCTGGACG |
| *Pgk1 rev* | GCAGCCTTGATCCTTTGGTTG |
| *Prl3d1 fwd* | TGGAGCCTACATTGTGGTGG |
| *Prl3d1 rev* | TGGCAGTTGGTTTGGAGGA |
| *Sdha fwd* | TGGTGAGAACAAGAAGGCATCA |
| *Sdha rev* | CGCCTACAACCACAGCATCA |
| *Tpbpa fwd* | CCAGCACAGCTTTGGACATCA |
| *Tpbpa rev* | AGCATCCAACTGCGCTTCA |
| *Ywhaz fwd* | TTGATCCCCAATGCTTCGC |
| *Ywhaz rev* | CAGCAACCTCGGCCAAGTAA |

**Table S2: Antibodies and lectins**

| Antibody | Source | Label | Manufacturer | Catalog no. | Dilution |
|---|---|---|---|---|---|
| Anti-Albumin | goat | - | Abcam | ab19194 | WB: 1:5000 |
| Anti-Actin | mouse | - | Millipore | #MAB1501 | WB: 1:100000 |
| Anti-Bovine IgG, IgM, IgA | rabbit | - | Thermo Fisher | SA1-36042 | IF: 1:100 |
| Anti-Cdx2 | rat | - | Abcam | ab76541 | IF: 1:300 |
| Anti-CD59 | rat | - | BioRad | MCA715G | HA: 2 µg/ml<br>CRA: 10 µg/ml |
| Anti-Cmas serum | rabbit | - | made in-house (4) | - | WB: 1:15000 |
| Anti-C3 | goat | - | MP Biomedicals | #55463 | WB: 1:5000<br>IF: 1:200 |
| Anti-Eomes | rabbit | - | Invitrogen | 14-4875-82 | IF: 1:100 |
| Anti-Factor H | goat | - | Complement Technology | A237 | IF: 1:1000<br>FC: 1:100 |
| Anti-Flag | rabbit | - | Cell Signaling | 2368 | WB: 1:5000 |
| Cholera Toxin B Subunit | vibrio cholera | FITC | Sigma-Aldrich | C9903 | IF: 1:200 |
| Cholera Toxin B Subunit | vibrio cholera | Alexa488 | Invitrogen | C34775 | FC: 1:400 |
| Anti-rat-IgG | goat | Alexa488 | Invitrogen | A11006 | IF: 1:500 |
| Anti-rabbit-IgG | sheep | Cy3 | Sigma-Aldrich | C2306 | IF: 1:500 |
| Anti-goat-IgG | donkey | Alexa488 | Invitrogen | A11055 | IF: 1:500 |
| Anti-goat-IgG | donkey | Alexa647 | Invitrogen | A 21447 | FC: 1:500 |
| Anti-goat-IgG | rabbit | HRP | Jackson | 305-035-003 | WB: 1:15000 |
| Anti-rabbit-IgG | goat | HRP | Jackson | 111-035-003 | WB: 1:15000 |
| Anti-mouse-IgG+IgA+IgM | goat | HRP | Southern Biotech | 1010-05 | WB: 1:15000 |

**References**


1. Christensen S, and Egebjerg J. Cloning, expression and characterization of a sialidase gene from Arthrobacter ureafaciens. *Biotechnology and applied biochemistry.* 2005;41(Pt 3):225-31.
2. Moreno-Indias I, Dodds AW, Argüello A, Castro N, and Sim RB. The complement system of the goat: haemolytic assays and isolation of major proteins. *BMC veterinary research.* 2012;8:91.
3. Neelamegham S, Aoki-Kinoshita K, Bolton E, Frank M, Lisacek F, Lütteke T, et al. Updates to the Symbol Nomenclature for Glycans guidelines. *Glycobiology.* 2019;29(9):620-4.
4. Schaper W, Bentrop J, Ustinova J, Blume L, Kats E, Tiralongo J, et al. Identification and biochemical characterization of two functional CMP-sialic acid synthetases in Danio rerio. *The Journal of biological chemistry.* 2012;287(16):13239-48.

**Full uncropped gels**

**Figure 1, B**

**Anti CMAS Western Blot**

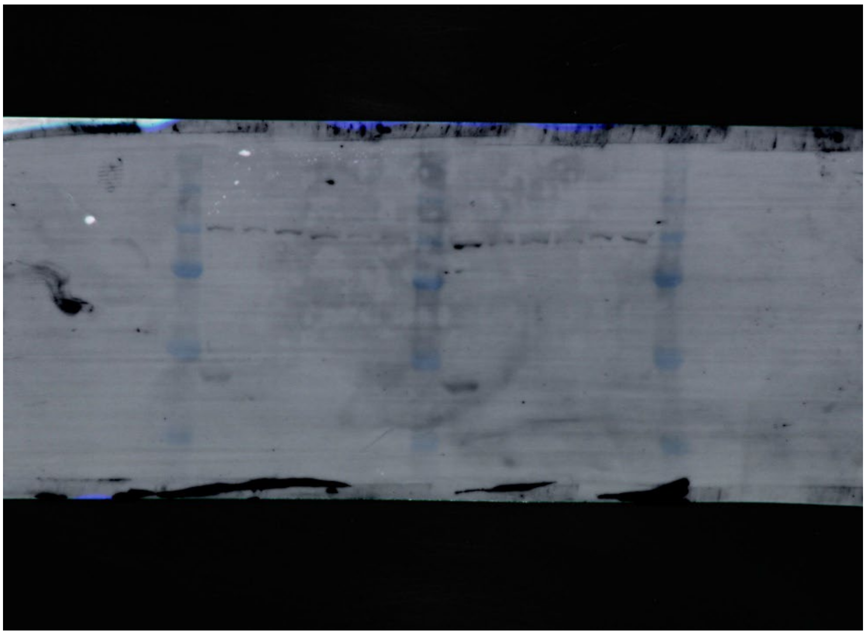

**Anti Actin**

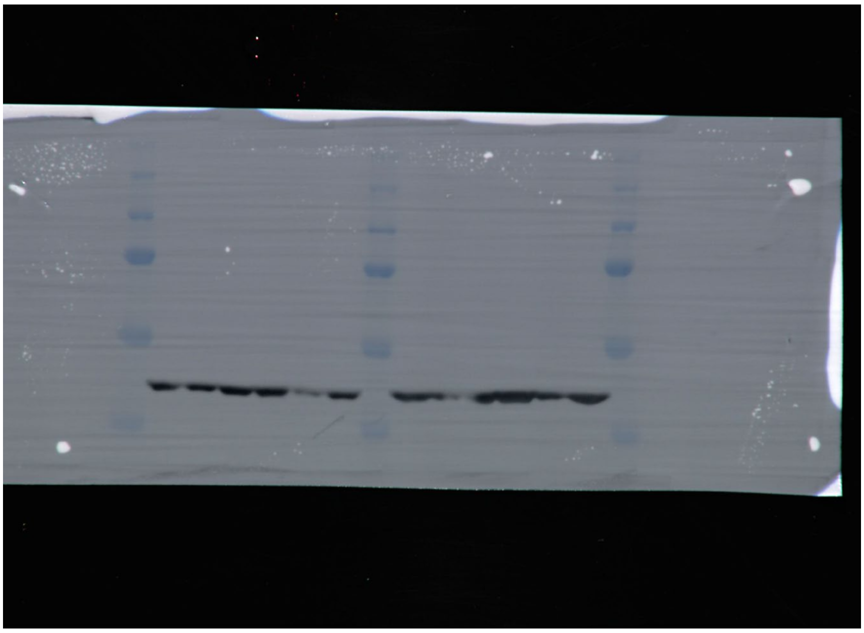

**Figure S3, A**

**Anti Flag**

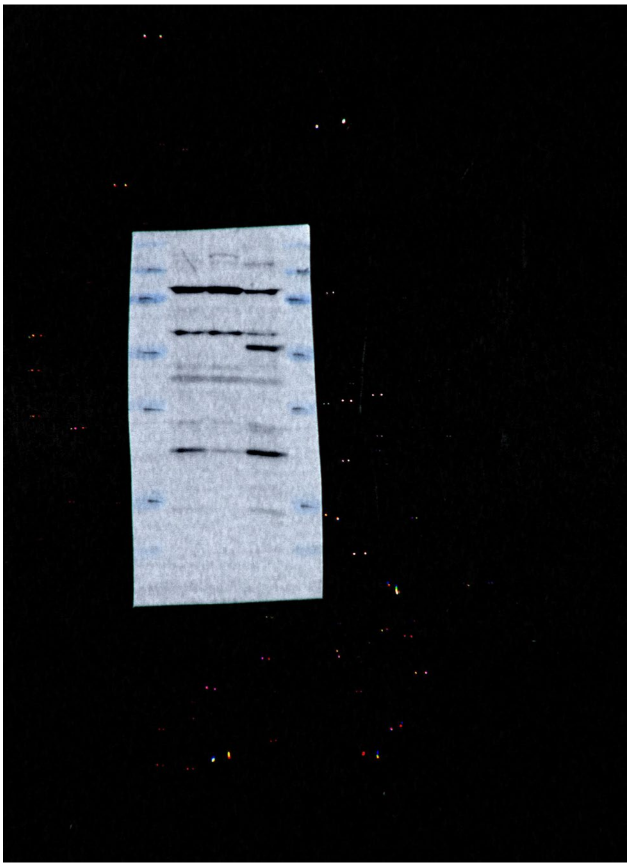

**Anti Actin** **Anti CMAS**

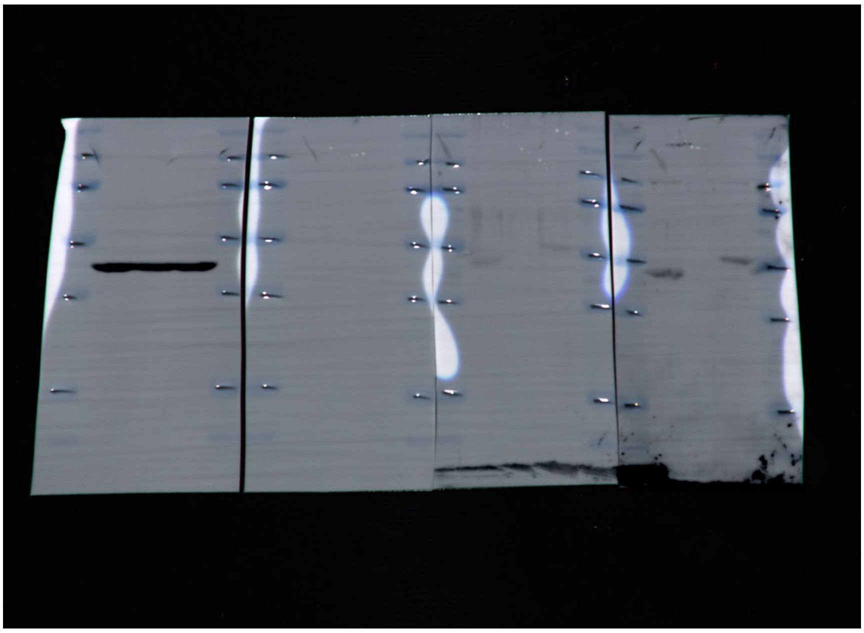

Supplement: Supplementary file 2 — Supplemental Material [file 41420_2023_1686_MOESM2_ESM.pdf]
